# The relationship between emotional intelligence, spiritual intelligence, and student achievement: a systematic review and meta-analysis

**DOI:** 10.1186/s12909-024-05208-5

**Published:** 2024-03-01

**Authors:** Zhenfei Zhou, Hamed Tavan, Forouzan Kavarizadeh, Mandana Sarokhani, Kourosh Sayehmiri

**Affiliations:** 1 Guangxi University of Foreign Languages , Nanning, China; 2 Psychosocial Injuries Research Center, Ilam University of Medical Sciences, Ilam, Iran; 3 Center for Educational Research in Medical Sciences (CERMS), Department of Medical Education, School of Medicine, Iran University of Medical Sciences, Tehran, Iran

**Keywords:** Student’s achievement, Emotional intelligence, Spiritual intelligence, Systematic review and meta-analysis

## Abstract

**Introduction:**

Emotional and spiritual intelligence are crucial factors in enhancing individuals’ knowledge and academic achievement. This study aims to examine the correlation between spiritual intelligence, emotional intelligence, and student achievement through a systematic review and meta-analysis.

**Materials and methods:**

A search was conducted in the PubMed, Scopus, Web of Science, SID, and Google Scholar databases from 2007 to December 2022. The effect sizes (EF) included the mean and standard deviation of emotional intelligence, spiritual intelligence, and student achievement and correlation coefficients among spiritual intelligence, emotional intelligence, and student achievement. Random effects models were used to pool the results, and the Q test and I^2^ index were employed to assess heterogeneity. Correlation coefficients were transformed into standard data (Z) using log transformation.

**Results:**

The overall mean score of educational achievement in university and school students was 15.91 (95% CI: 15.26–16.78). The mean scores of spiritual and emotional intelligence were 138.27 (95% CI: 129.19-147.35) and 128.94 (95% CI: 117.08–140.80), respectively. The correlation coefficients between spiritual intelligence, emotional intelligence, and student achievement were *r* = 0.36 (95% CI: 0.18–0.51) and *r* = 0.50 (95% CI: 0.28–0.67), respectively.

**Conclusions:**

Emotional and spiritual intelligence are independent predictive factors in educational achievement for university and school students. Therefore, improvements in emotional and spiritual intelligence can promote students’ academic achievement.

## Introduction

Education plays a key role in the success and prosperity of individuals. Student achievement is very important in success at work. Achievement is a multidimensional subject that can include the physical, social, cognitive, emotional, and spiritual growth of students [1]. The construct of intelligence is one of the most important factors in predicting achievement. Many studies have been conducted on intelligence and intelligence tests by psychologists. Additionally, many theories and definitions have been provided [2, 3]. However, there has been no consensus theory, as intelligence is an invisible and qualitative entity [3, 4]. In addition, intelligence itself is not measurable, and instead, its footprints can only be evaluated [5, 6]. Emotional intelligence includes the appropriate knowledge, management and expression of emotions. The interpersonal and intrapersonal parameters of emotional intelligence are categorized into five groups: 1- self-awareness, 2- the control of emotions (including the ways of managing and controlling emotions, feelings, fears, and anger), 3- self-motivation (directing and guiding emotions and thrills toward the aim and delaying demands and preventing efforts), 4- common sense (being sensitive regarding others’ interests and tolerating their opinions and granting the differences among people feelings), and 5- managing relations (concerned with controlling others’ thrills and having communicative skills) [7, 8].

Spiritual intelligence is a combination of spiritual abilities and interests, personal characteristics, special cognitive capabilities, and psychological processes. Spiritual intelligence is helpful in resolving existential and supernatural concerns [9, 10]. Spiritual intelligence comprises four abilities. First, existential and critical contemplation involves reflecting on issues such as life, death, afterlife, reality, justice and other existential or supernatural matters. Second, the ability to develop an individualized concept refers to creating personal purposes based on mental experiences and living according to those designed aims. Third, transcendental knowledge is the capacity to recognize transcendental and supernatural dimensions within oneself, others, and the universe. Last, extending knowledge entails the ability to enter to access spiritual or higher consciousness areas [11]. Psychologists have identified eight parameters for spiritual intelligence. These parameters include 1- patience, 2- spiritual and religious beliefs and actions, 3- life meaning and purpose, 4- priority, 5- internal peace, 6- spiritual experiences, 7- self-recognition, and 8- forgiveness [12, 13].

Students constitute a large part of our society. These individuals will become future drivers of societies [14]. Emotional and spiritual intelligence can be used as boosters of academic achievement in university and school students. It is possible to augment academic performance and status, as well as education quality, in students by implementing workshops to escalate different types of intelligence and their applications. Academic achievement in university students represents the success of the educational system of universities as an important goal [15, 16].

Meta-analysis studies aim to unite different opinions on a certain issue [17]. Therefore, it seems necessary to conduct meta-analysis studies to validate research outcomes and to guide researchers and policy makers. The aim of this study is to estimate correlation coefficients among spiritual intelligence, emotional intelligence, and student achievement using systematic review and meta-analysis.

## Materials and methods

This systematic review and meta-analysis was conducted following the PRISMA guidelines.

### Eligibility criteria

This is a systematic review and meta-analysis of studies published from 2007 to December 2022. Those studies related to emotional or spiritual intelligence and their relationships with educational achievement among university students and high school students.

We selected the studies used EQ-i 2.0 (Emotional Quotient Inventory), MSCEIT (Mayer-Salovey-Caruso Emotional Intelligence Test) and EISA (Emotional Intelligence Skills Assessment) for assessment emotional intelligence, SQ-5R (Spiritual Quotient 5R Inventory), SAI (Spiritual Assessment Inventory) and SSI (Spiritual Intelligence Scale) for assessment spiritual intelligence, standardized tests or classroom assessments for assessment student achievement.

Articles published in English or Persian were included in the study. The exclusion criteria were as follows: studies that did not report the mean, standard deviation, correlation coefficients, or sample size. Short communication articles were excluded. Not meeting qualification criteria, being unrelated to the research question, incomplete data, and abstracts presented in congress lacking enough information and inaccessibility of full texts were considered exclusion criteria.

Studies were included that reported the mean and standard deviation of emotional intelligence, spiritual intelligence, and student achievement, as well as correlation coefficients among emotional intelligence, spiritual intelligence, and student achievement.

### Search strategy

Two researchers independently performed a literature search in Magiran, Iran Medex, SID, Iran Doc, Scopus, PubMed, Science Direct, and Web of Science databases, as well as the Google Scholar search engine. Based on the study aims, the keywords included MeSH terms: “spiritual intelligence”, “emotional intelligence”, and “academic achievement”. To perform a comprehensive search, Boolean operators (AND, OR, NOT) were applied to conduct combined searches. Studies assessing the relationship between these entities among Iranian university students were included. In parallel, the literature search was also performed in Iranian databases. Additionally, related studies were also obtained by screening reference lists of the selected articles. These newly found articles were assessed if they were not duplicates.

### Selection process

The titles and abstracts of 124 articles were read by Zhou and Hammed. Based on the screening of the title and abstract, the decision was made on whether to include the studies in the review. To minimize bias, the articles were independently selected and extracted by two researchers. In case of disagreements, the final decision regarding the inclusion or exclusion of the studies was made by a third researcher. The relevant articles were selected by two researchers.

### Data collection process

Two researchers independently performed data extraction in each article. The following data were extracted from the articles: sample size, mean and standard deviation of emotional intelligence, spiritual intelligence, and student achievement, as well as correlation coefficients among emotional intelligence, spiritual intelligence, and student achievement. The articles were not translated from Persian to English or from English to Persian, as the researchers had a good understanding of the articles and were able to perform the data extraction.

### Data items

In this study, the effect sizes (EF) were calculated as correlation coefficients between spiritual intelligence, emotional intelligence, and student achievement. Since correlation coefficients do not follow a normal distribution, a log transformation was applied to the data.

Other data items included the first author’s name, the date and location of the study, the sample size, the means of emotional and spiritual intelligence scores, the mean academic achievement scores, the educational group (university or school), and the country.

### Study risk of bias assessment

Quality assessment of the studies was conducted using the Joanna Briggs Institute( JBI ) Critical Appraisal Checklist. All studies included in the meta-analysis process were deemed to have acceptable quality.

### Effect measures

The effect size was a continuous variable. The main effect sizes (EF) were correlation coefficients among spiritual intelligence, emotional intelligence, and student achievement. The range of the correlation coefficient is 0 ≤ *r* ≤ 1.

### Synthesis methods (preparing for synthesis)

The main index for student’s achievements was average scores. In two studies, the average scores were originally measured out of 30, but they were later converted to a scale out of 20.

### Synthesis methods (tabulation and graphical methods)

In the study characteristics for individual studies, the author’s last name, year of publication, place of research, total sample size, sample size for boys and girls, mean and standard deviation of student achievements, spiritual intelligence, and emotional intelligence were included.

Forest plots, was utilized for graphical presentation of the studies.

### Synthesis methods (statistical synthesis methods)

The correlation coefficient is between$- 1 \leqslant r \leqslant 1$, and its distribution is not normal. To normalize the correlation coefficient, link $z = \frac{1}{2}\ln \left( {\frac{{1 + r}}{{1 - r}}} \right)$ was employed. The standard deviation of the z score was estimated using the $SE = \frac{1}{{\sqrt {n - 3} }}$ formula. Meta-analysis was conducted on z scores, and the resulting z values were then transformed into R-values (correlation coefficients) using the $r = \frac{{{e^{2Z}}}}{{1 + {e^{2Z}}}}$formula.

Heterogeneity among the studies was assessed using the Q test and I^2^ index. The heterogeneity among the studies was found to be significant; therefore, a random effects model was used to pool the data. The heterogeneity index of the present study was determined to be 94.5%, indicating a high degree of heterogeneity [18, 19].

Meta-regression was applied to check for heterogeneity reasons and to assess a potential link between emotional intelligence, spiritual intelligence, and educational achievement in individual years. Subgroup analysis was conducted for high school students and university students. The statistical methods were performed using STATA software (version 15).

## Results

### Study selection (flow of studies)

**Fig. 1:**
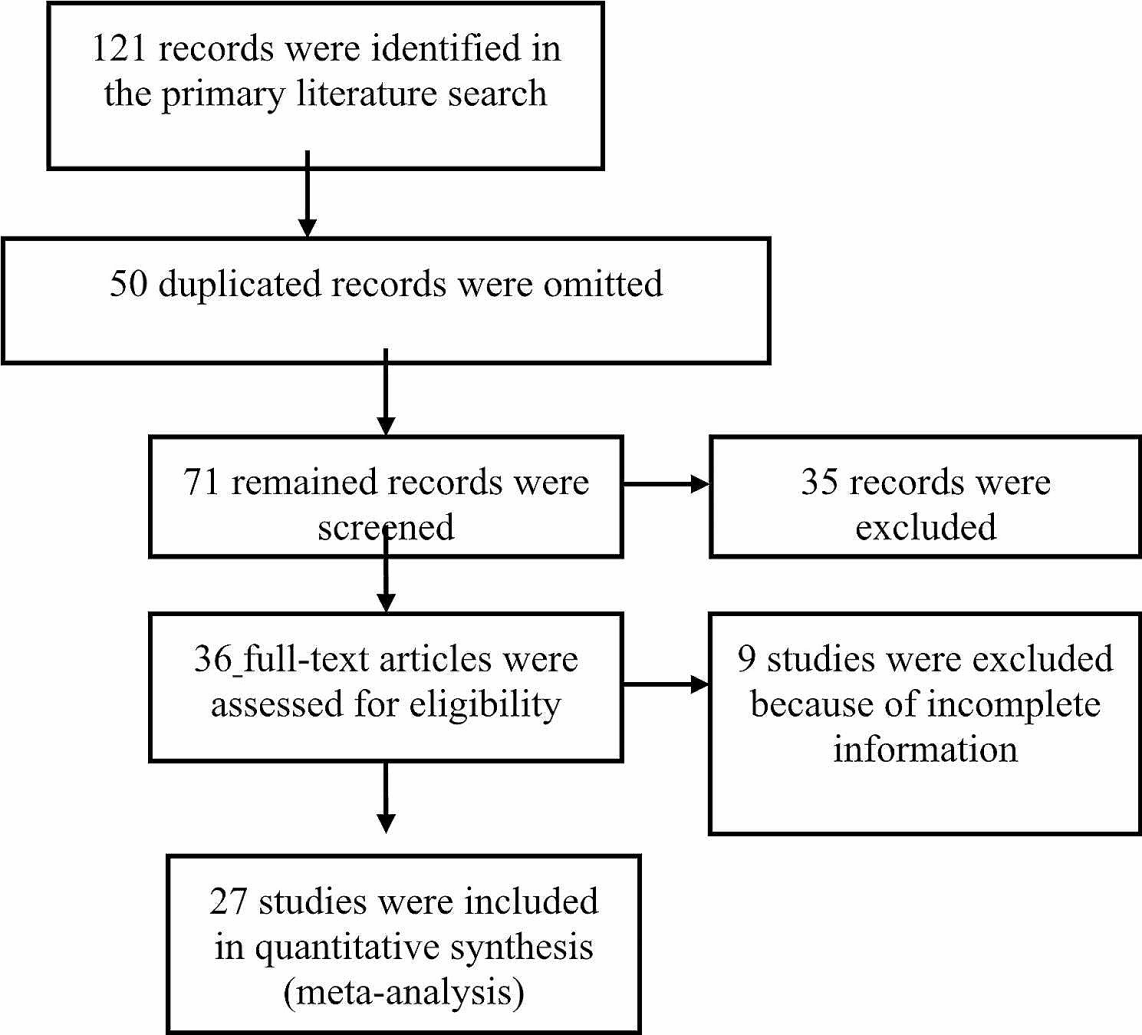
Flowchart of the present systematic review and meta-analysis

One hundred twenty-one articles were recruited in the primary search. Two of the researchers independently evaluated titles and abstracts. In case of being related to the research question, the full texts were obtained. After reading titles, abstracts, and full texts, 50, 35, and 9 studies were omitted because they were duplicated, unrelated, or had inadequate information, respectively. Ultimately, 27 articles with the desired qualifications were selected for systematic review (Fig. 1).

### Study characteristics

The final studies were conducted between 2007 and 2022, with a total sample size of 5781 students, including 1910 high school students and 3871 university students. In six study with 535 sample they did not report sample according to male and female. The characteristics of the studies are represented in Table 1.

**Table 1:** The study characteristics

Reference number*	First author’s	Year	country	Sample size	Sample size in female	Sample size in male
[20]	Arbabisarjou	2013	Iran	250	118	132
[21]	Dastjerdi	2017	Iran	150	100	50
[22]	Dashtbozorgi	2016	Iran	300	180	120
[23]	Omidvar	2014	Iran	288	144	144
[24]	Molazadeh	2013	Iran	338	195	143
[25]	Hanifi	2010	Iran	380	180	200
[26]	Keshavarz	2014	Iran	100	69	31
[27]	Amiri	2016	Iran	145	124	21
[28]	Raeesi	2013	Iran	353	241	112
[29]	Samari	2007	Iran	112	56	56
[30]	Hosaeeninasab	2008	Iran	315	161	154
[31]	Safarian	2013	Iran	180	117	63
[32]	Ashoori	2013	Iran	400	215	185
[33]	Farahangpour	2010	Iran	357	207	150
[34]	Loghmanpour	2017	Iran	624	312	312
[35]	Rahmawati	2019	Indonesia	150.0	-	-
[36]	Rajeswari	2019	India	297.0	104.0	193
[37]	Turi	2020	Pakistan	113.0	-	-
[38]	Zaheri	2020	Iran	125.0	-	-
[39]	Khan	2019	India	80.0	-	-
[40]	Ibrahim	2022	Malaysia	157	104	53
[41]	Fallahzadeh H	2011	Iran	223	153	70
[42]	Sanchez	2020	Spain	44	-	-
[43]	Ranjbar	2017	Iran	23	-	-

*Numbers indicate the reference number in the references list.

Of the total sample, 3038 were female, and 2090 were male. The mean age was 19.9 ± 2.1. Table 2 presents the mean and standard deviation of spiritual intelligence, emotional intelligence, and student achievement in male and female students.

**Table 2:** Mean and standard deviation of spiritual intelligence, emotional intelligence, and student achievement in male and female students

		Mean	Standard Deviation
Spiritual Intelligence	male	143.24	19.96
	female	146.39	18.30
Emotional Intelligence	male	117.18	17.10
	female	118.42	16.2
Students Achievement	male	16.48	1.15
	female	17.1	1.18

**Fig. 2:**
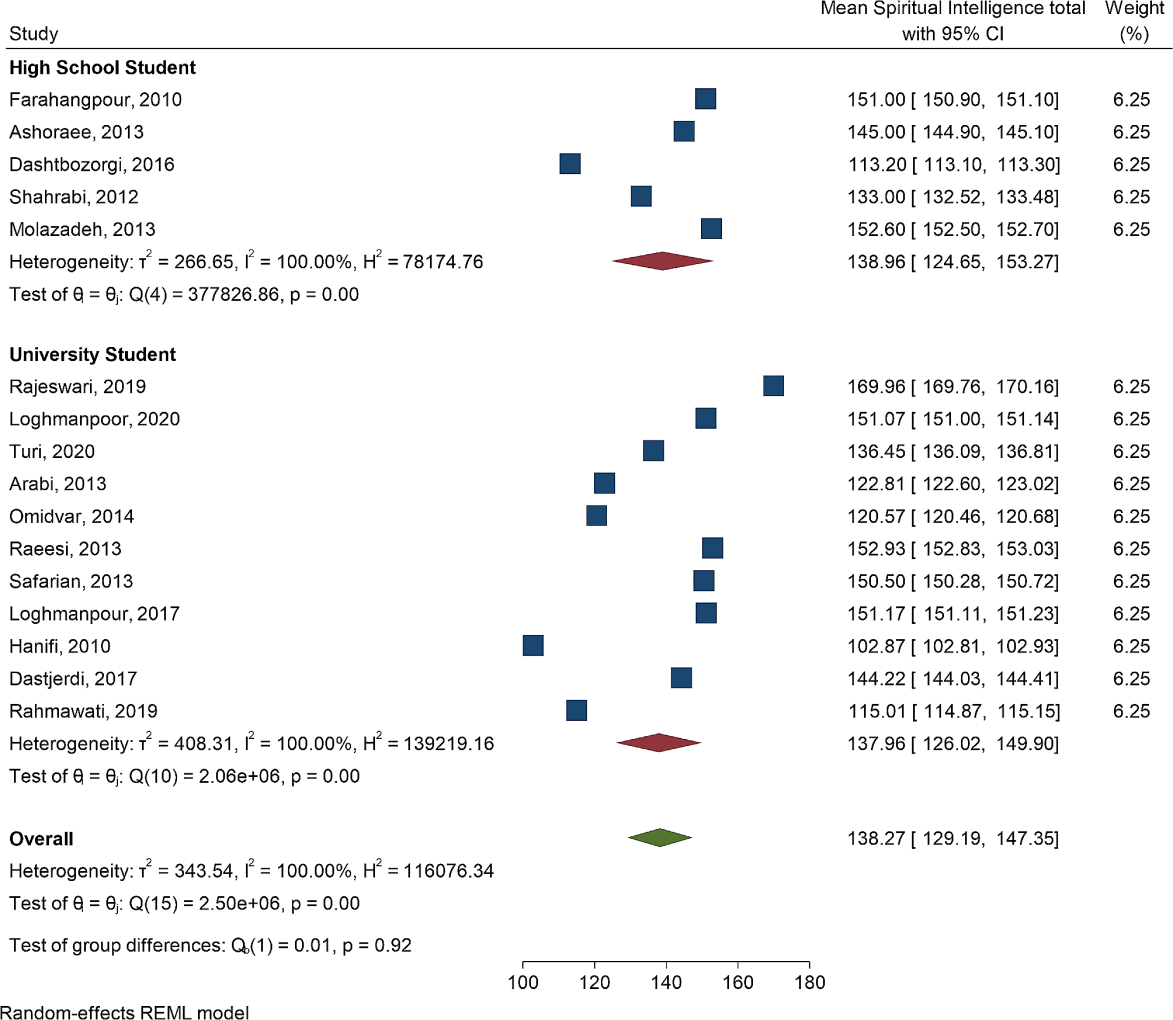
Forest plot mean of spiritual intelligence according to high school students and university students

**Fig. 3:**
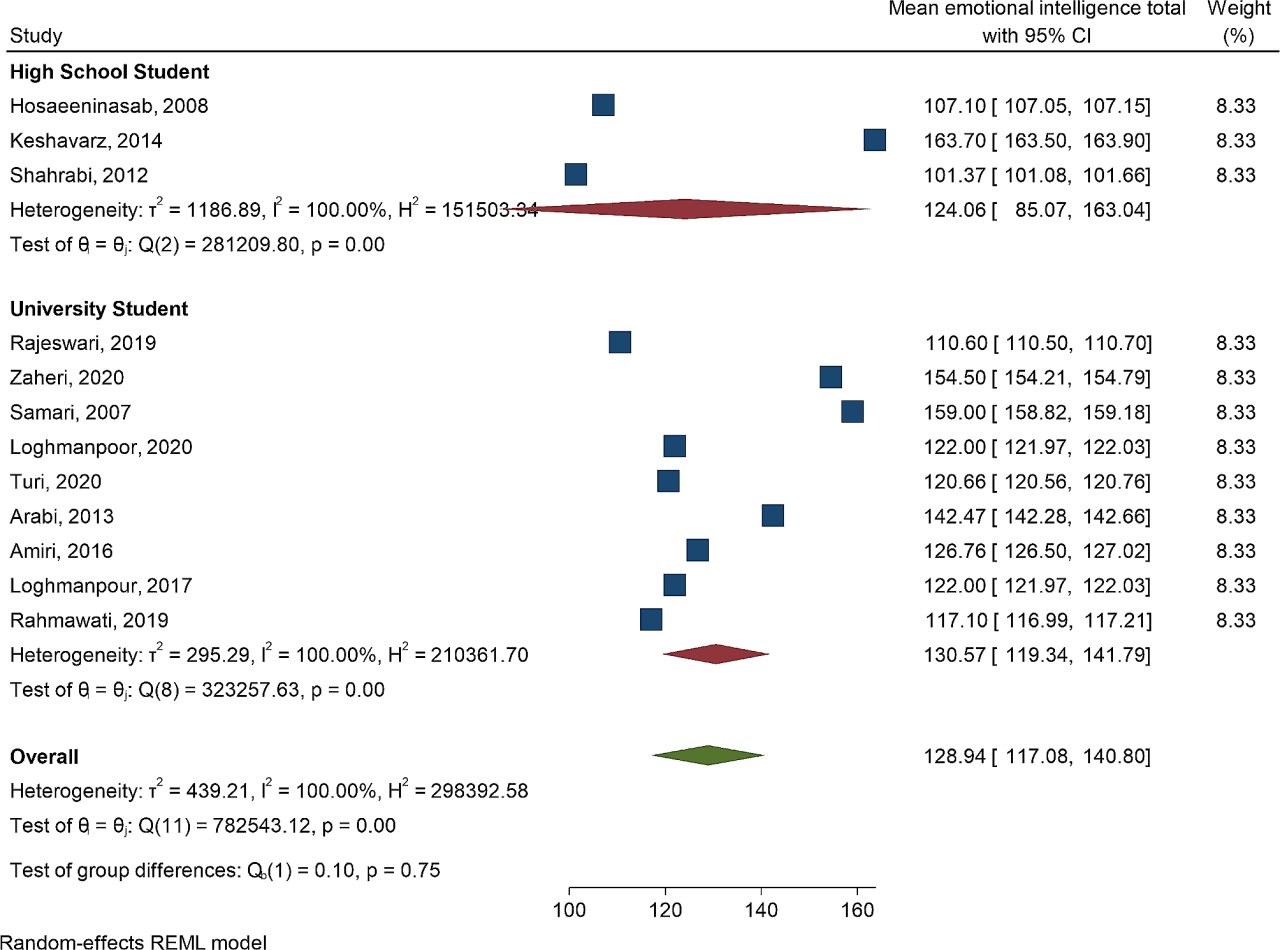
Forest plot mean of emotional intelligence according to high school students and university students

**Fig. 4:**
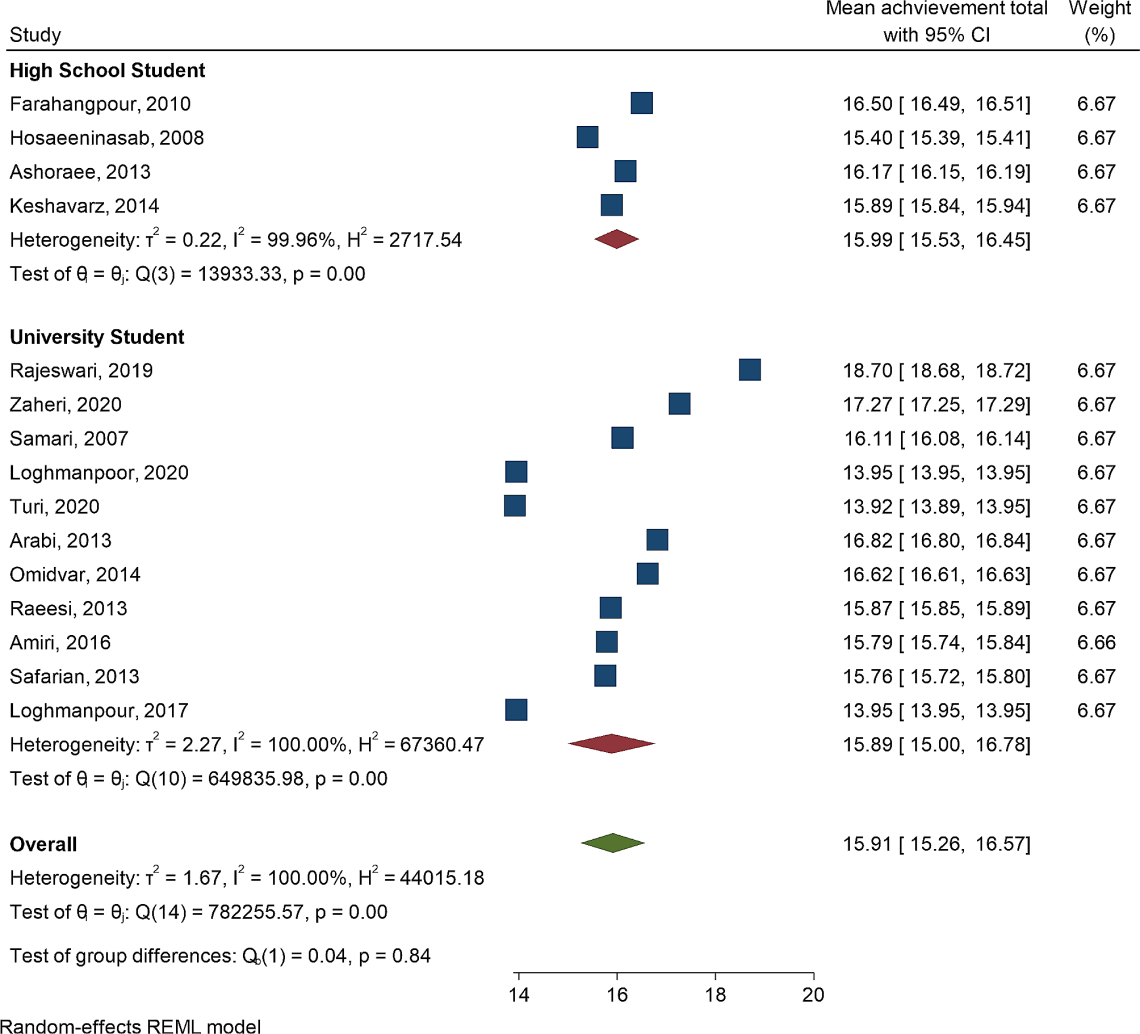
Forest plot mean of achievements according to high school students and university students

Figures 2 and 3, and 4 display the mean and 95% confidence interval of spiritual intelligence, emotional intelligence, and academic achievement for both high school students and university students. In Fig. 2, the means and 95% confidence intervals of spiritual intelligence for high school students and university students indicate no significant difference between the two groups (*P* = 0.92). However, there was significant heterogeneity among the studies (I^2^ = 100, *P* < 0.001.

Figure 3 demonstrates that there is no significant difference in the means of emotional intelligence between high school students and university students (*P* = 0.75). However, there was significant heterogeneity among the studies (I^2^ = 100, *P* < 0.001.

**Fig. 5:**
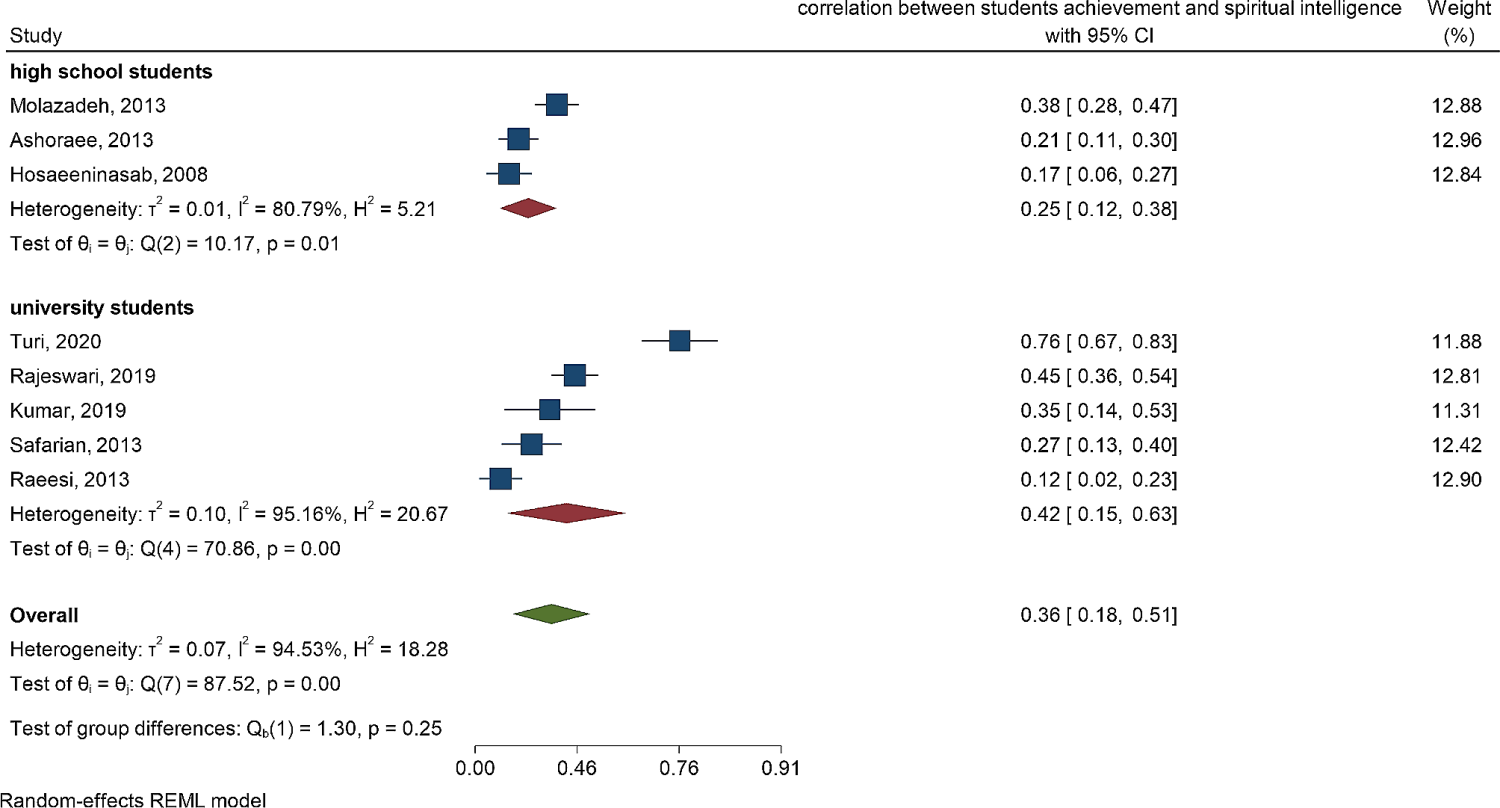
Forest plot correlation between student’s achievement and spiritual intelligence according to high school students and university students

**Fig. 6:**
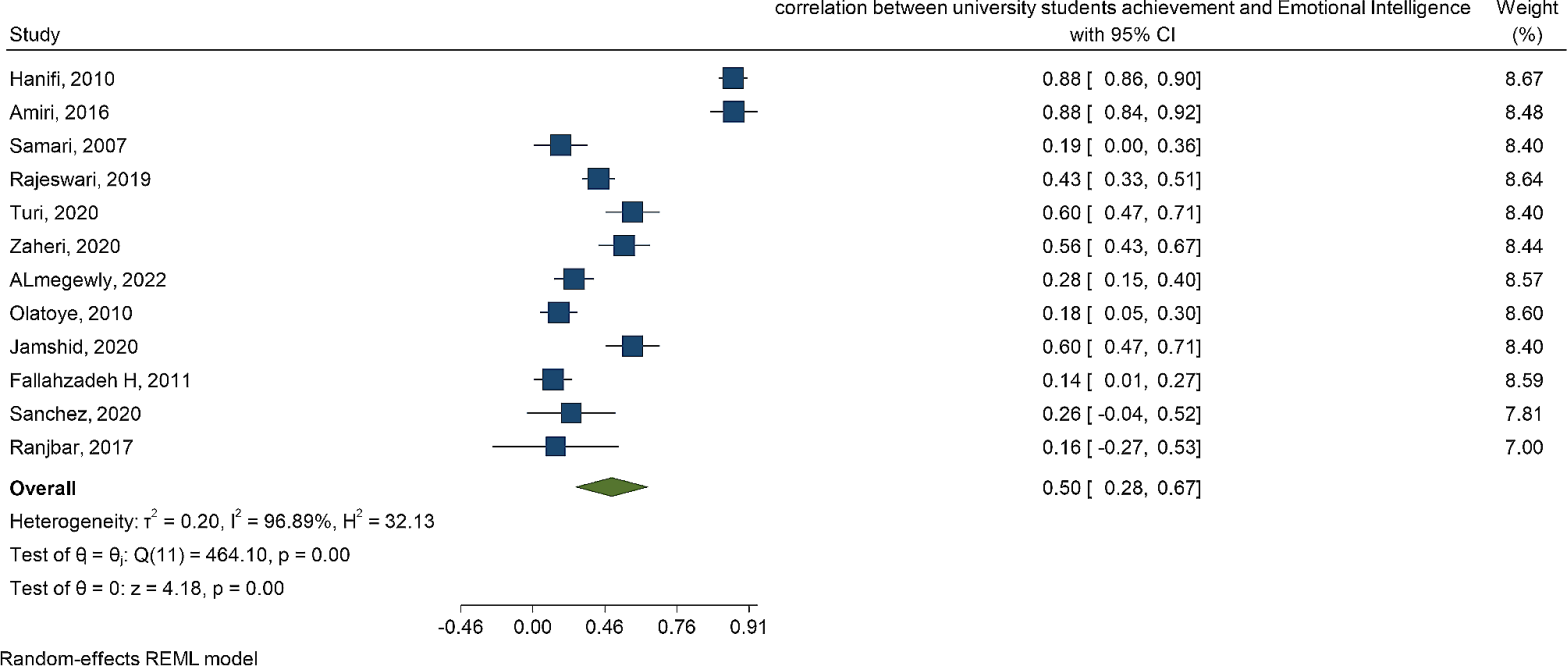
Forest plot correlation between student’s achievement and emotional intelligence in university students

The means and 95% confidence intervals of spiritual intelligence for high school students and university students indicate no significant difference between the two groups (*P* = 0.92). Additionally, there was significant heterogeneity among the studies (I^2^ = 100, *P* < 0.001). Similarly, the test of group difference in Fig. 4 reveals no significant difference in student achievement between high school students and university students (*P* = 0.82). Again, there was significant heterogeneity among the studies (I^2^ = 100, *P* < 0.001). Figures 5 and 6 display the forest plot correlation between student achievements and spiritual intelligence, as well as emotional intelligence.

The correlation coefficients between spiritual intelligence and student achievement were *r* = 0.25 and *r* = 0.42 for high school students and university students, respectively (Fig. 5). This indicates that the correlation between spiritual intelligence and student achievement is lower in high school students than in university students.

The correlation coefficients between spiritual intelligence and student achievement were *r* = 0.25 and *r* = 0.42 for high school students and university students, respectively (Fig. 5). This shows that the correlation between spiritual intelligence and student achievement is lower in high school students than in university students. In the analysis of 8 studies, the correlation between student achievement and emotional intelligence was estimated. By pooling the results, the correlation between student achievement and emotional intelligence was estimated to be *r* = 0.50 (Fig. 6).

## Discussion

The aim of the present study was to assess the relationship between spiritual intelligence, emotional intelligence, and student achievement among university students and high school students through a systematic review and meta-analysis. The results of this study indicated that spiritual intelligence, emotional intelligence, and student achievement were similar among high school students and university students, with no significant differences observed in these three measures. The correlation coefficient between spiritual intelligence and student achievement, as well as the correlation coefficient between emotional intelligence and student achievement, were found to be significant. However, emotional intelligence emerged as a stronger predictor of student achievement than spiritual intelligence.

The results of studies investigating the relationship between intelligence and different educational grades varied [24–28]. Based on these divergent results, it can be concluded that higher spiritual intelligence in both university and school students may have significant impacts on their academic status. The present study revealed that school students had a slightly higher mean score of spiritual intelligence compared to university students, although the difference was not statistically significant. This difference may be partly attributed to the higher age, increased concerns with life problems, and occupational perspectives among university students [20, 22, 29–31]. Additionally, girls had a higher mean score of spiritual intelligence than boys, which aligns with previous studies [21, 23, 28]. On the other hand, university students exhibited a higher mean score of emotional intelligence compared to school students. This may be attributed to the fact that university students are mature individuals who have experienced various life events [24–29]. Although girls had a higher mean emotional intelligence score than boys, this difference was not statistically significant (*P* > 0.05). These observations are consistent with the findings of previous studies [26–29].

When considering educational grades, academic achievement showed a higher mean score in school students than in university students. This difference may be attributed to the varying levels of education in universities and schools, where larger educational content is covered in relatively shorter periods in universities [26–28, 38]. In research, there was a positive and significant relationship between emotional intelligence (i.e., self-awareness, social awareness and emotional receptivity) and spiritual intelligence [40]. This study was conducted in learning organizations to see the impact of emotional and spiritual intelligence on academic performance. The findings provide positive and significant correlations among the types of intelligence and academic performance [37]. Additionally, Pearson’s correlation coefficient showed that there is a significant (*r* = 0.14, *p* = 0.039) relationship between emotional intelligence and academic performance, while the findings indicated a meaningful relation (*p* < 0.05) between its two subcomponents, emotional intelligence and academic performance [41]. In the study conducted by Mr. Sanchez and colleagues, it was concluded that emotional intelligence affects academic performance and that there is a positive relationship [42]. In a study conducted by Ranjber et al., a weak correlation was observed between emotional intelligence and educational achievement in the context of Iranian university students [43]. The mean academic achievement score was also higher among girls than boys; however, this difference was not statistically significant (*P* > 0.05). This finding is consistent with previous reports, which may be attributed to the boys’ responsibilities and part-time jobs. On the other hand, girls tend to spend more time studying and learning, which contributes to higher levels of academic achievement compared to boys. The correlation coefficients indicate that both spiritual and emotional intelligence are important factors in the academic achievement of university and school students. Implementing workshops on the application of various types of intelligence could potentially enhance academic performance in students and improve the quality of educational programs.

Emotional intelligence affects self-efficacy and the attainment of social acceptance, both of which positively influence student achievement [32, 36, 44]. Many researchers consider emotional intelligence to be the most important predictor of student achievement [33, 34, 45]. Our findings align with those of several researchers, indicating a significant relationship between emotional intelligence and student achievement [35, 39, 46, 47]. However, our findings contradict the findings of Ademola et al [47].

The results of this meta-analysis demonstrated that spiritual intelligence is one of the most important factors for improving students’ achievement. Spiritual intelligence can enhance students’ performance and contribute to their success in education. It appears that students with higher levels of spiritual intelligence adapt better to different educational methods than those with lower levels. Therefore, incorporating strategies that cater to the needs of students with varying levels of spiritual intelligence can lead to more successful educational outcomes.

### Limitations

The limitations of this study include the fact that the majority of research on the correlation coefficient among spiritual intelligence, emotional intelligence, and student achievement was conducted in Islamic countries, with many of these studies originating from Iran. Additionally, some studies did not report correlation coefficients, while others did not report correlation coefficients according to gender and grade.

Recommendation: According to our results, both spiritual and emotional intelligence are important factors influencing academic achievement among Iranian students. Holding related workshops is a viable option to boost academic achievement among school and university students.

## Conclusion

This systematic review and meta-analysis study estimates the correlation between spiritual intelligence, emotional intelligence, and student achievement among high school students and university students. The results of the present study demonstrate a positive and significant correlation between spiritual intelligence, emotional intelligence, and student achievement (*p* < 0.001).

## Data Availability

This is a meta-analysis; data are available directly from Table 1; Figs. 2, 3, 4, 5 and 6 of this article. The data used and analyzed during the current study are available from the corresponding author upon reasonable request.
